# Random Finite Set Based Parameter Estimation Algorithm for Identifying Stochastic Systems

**DOI:** 10.3390/e20080569

**Published:** 2018-07-31

**Authors:** Peng Wang, Ge Li, Yong Peng, Rusheng Ju

**Affiliations:** College of Systems Engineering, National University of Defense Technology, Changsha 410073, China

**Keywords:** parameter estimation, importance sampling, Probability Hypothesis Density (PHD), Random Finite Set (RFS), Markov Chain Monte Carlo (MCMC), simulated tempering

## Abstract

Parameter estimation is one of the key technologies for system identification. The Bayesian parameter estimation algorithms are very important for identifying stochastic systems. In this paper, a random finite set based algorithm is proposed to overcome the disadvantages of the existing Bayesian parameter estimation algorithms. It can estimate the unknown parameters of the stochastic system which consists of a varying number of constituent elements by using the measurements disturbed by false detections, missed detections and noises. The models used for parameter estimation are constructed by using random finite set. Based on the proposed system model and measurement model, the key principles and formula derivation of the proposed algorithm are detailed. Then, the implementation of the algorithm is presented by using sequential Monte Carlo based Probability Hypothesis Density (PHD) filter and simulated tempering based importance sampling. Finally, the experiments of systematic errors estimation of multiple sensors are provided to prove the main advantages of the proposed algorithm. The sensitivity analysis is carried out to further study the mechanism of the algorithm. The experimental results verify the superiority of the proposed algorithm.

## 1. Introduction

In modern society, mathematical models are playing increasingly important roles in engineering systems when used for simulating, predicting, managing, and deciding [1]. Since system identification is essential for modeling, it is the potential and vital technology to meet the challenges of model based scientific research. System identification has been applied to many areas, including industrial systems, agricultural systems, biological systems, medical systems and economic systems, and even social systems [2,3,4,5].

Generally speaking, the existing system identification algorithms can be classified into non-parametric model identification algorithms and parametric model identification algorithms [6]. Parametric models are more powerful, because they can represent the studied system with fewer parameters and have more statistical power. The parametric model identification algorithms usually assume some model structure and estimate the associated parameters by reducing the error criterion function of the model and practical system. Therefore, parameter estimation is the core component of these algorithms.

### 1.1. Parameter Estimation

Parameter estimation refers to identifying, estimating and calibrating continuous or discrete model parameters [7]. The parameters to be estimated could be the time-invariant physical quantities, or the coefficients and other constants in a functional relationship that describe the practical physical system [8]. They can be represented by scalars, vectors, or matrixes. In this paper, we use an *n* elements vector θ∈Θ to represent the unknown model parameters.

The stochastic system is the system whose input, output and interference have random factors, or the system itself has some uncertainty. Only by parameter estimation can we get the model parameters for the stochastic system. Parameter estimation of the stochastic system is the basis of state filtering, stochastic control, fault diagnosis, forecasting and many other fields [9]. This paper deals with the identification problem of stochastic systems whose model structures are known and parameters to be identified. In this case, the identification of stochastic system minimizes the estimating of unknown parameters associated with the known models by using prior information and observations from the practical system.

The Sequential Monte Carlo (SMC) method is a powerful tool to perform optimal state and parameter estimating by using the nonlinear and non-Gaussian state space models. Since SMC based parameter estimation algorithms employ the non-parametric statistic inference based on Bayesian inference theory, and have no assumptions of the distribution and linearity of the studied system, they are widely used for parameter estimation [10,11,12,13,14].

According to [11], the SMC based parameter estimation algorithms can be broadly formulated into Maximum Likelihood (ML) formulation and Bayesian formulation. ML formulation is mainly related to finding a point estimate of the unknown parameter which is the most likely to be associated with the observations. The ML estimate of the parameters is the parameters that minimize the negative logarithm of the likelihood function based on the received measurements. The estimation process is shown as the following equation:

(1) $${\hat{\theta }}_{ML}=\arg\max_{\theta \in \Theta }p_{\theta }\left(z_{1:T}\right)=\arg\max_{\theta \in \Theta }\log p_{\theta }\left(z_{1:T}\right)$$

where ${\hat{\theta }}_{ML}$ is the estimated parameter vector, $z_{1:T}$ is the sequence of measurements, and θ is the unknown parameter vector to be estimated.

ML method is only applicable to the estimation problem where a joint probability density function (PDF) can be assigned to the measurements [15]. In addition, ML estimate needs to use the gradient based search methods such as a damped Gauss–Newton method to actually compute estimated parameters [16].

In the Bayesian formulation, the unknown parameters θ are regarded as an unobserved random variable or vector. The Bayesian parameter estimation gives a posterior PDF of the unknown parameters, conditioned on measurements and prior distribution. Thus, the parameter estimation problem refers to obtaining the posterior PDF of θ by using the available measurements. According to Bayes’ theory, the posterior distribution can be obtained by Equation (2), where $p_{0}\left(\theta \right)$ is the prior distribution.

(2) $$p\left(\theta |z_{1:T}\right)=\frac{p\left(z_{1:T}|\theta \right)p_{0}\left(\theta \right)}{p(z_{1:T})}$$

The first advantage of the Bayesian parameter estimation algorithm over the traditional algorithms is the conceptualization of the estimation result. In the traditional algorithms, there is a specific but unknown model that needs to be identified. In the Bayesian algorithm, the model is a random variable rather than deterministic. The estimation result is a probability distribution for the model parameters named as posterior distribution. With the probability distribution, we can answer many probabilistic questions about the model. Such questions are impossible in the traditional algorithms, because the identified model they provided is not a random variable.

The second advantage of the Bayesian parameter estimation algorithm over the traditional algorithms is the incorporation with prior information represented mathematically as a prior distribution for the model. The Bayesian parameter estimation algorithm naturally incorporates prior information about the estimation result, ranging from hard additional constraints to experience based intuition. Once measurements are collected, they are combined with the prior distribution using Bayesian inference to produce the desired posterior distribution of the unknown parameters.

Both ML and Bayesian parameter estimation can be implemented off-line or on-line. In an off-line parameter estimation algorithm, we infer the unknown parameters by iterating over a fixed measurement dataset. In an on-line method, the unknown parameters are sequentially estimated as the measurements become available. The role of SMC for both off-line and on-line parameter estimation is discussed in [10,17]. If we assume that the states of the studied system are unknown, there is a need to deal with the relationship between states and parameters. Marginalization and data augmentation are two different strategies for dealing with this kind of parameter estimation problem. Marginalization means integrating out the states from the problem and viewing the unknown parameters as the only unknown variable of interest. Usually, marginalization is implemented in an off-line manner. Data augmentation estimates the unknown parameters together with the states [11,18]. In other words, the data augmentation performs parameter estimation by extending the state with the unknown parameters and transforming the problem into an optimal filtering problem. Usually, data augmentation is implemented in an on-line manner.

### 1.2. Nomenclature

An introduction of some concepts used in this paper is provided in the following. More detailed information can be found in the provided references.

#### 1.2.1. Random Finite set (RFS)

In the middle 1970s, G. Matheron systematically described the random set theory [19]. The random finite set (RFS) approach introduced by Mahler is an advanced theory that unifies much of information fusion under a single Bayesian paradigm [20,21]. The RFS ψ is defined as a random variable that draws its instantiations ψ=Y from the hyperspace X of all finite subsets *Y* (the null set ∅ included) of some underlying space $X_{0}$. Here, we assume that $X_{0}=R^{n}$ is a Euclidean vector space and that, therefore, X consists of all finite sets of the form Y=∅ or $Y=\{y_{1},\ldots ,y_{n}\}$ where n≥1 is an arbitrary positive integer and $y_{1},\ldots ,y_{n}$ are arbitrary vectors in $R^{n}$.

#### 1.2.2. Finite Set Statistics (FISST)

Here, we adopt FISST which provides the mathematical representation and manipulation of RFS. FISST is a set of tools for filtering and estimating problems involving RFS [22]. FISST enables the RFS based Bayesian inference by providing some mathematical tools. Fundamentally, it approaches the modeling of multi-object tracking by introducing sets that contain a random number of single-object states, each of which is a random vector. It allows the dynamics of each object (member of the object set) to vary according to some motion model while the number of objects (members in the set) is allowed to vary according to some point process model.

#### 1.2.3. Probability Hypothesis Density (PHD)

The difficulty of RFS based Bayesian inference is its computational complexity. To make the RFS based estimation possible, Mahler proposed the PHD (probability hypothesis density) filter [21,23,24]. The PHD $D_{k|k}\left(x|Z_{k}\right)$ of the posterior probability PDF $f_{k|k}\left(X_{k}|Z_{k}\right)$ is a density function defined on the single object state $x\in X_{0}$ as follows:

(3) $$D_{k|k}\left(x|Z_{k}\right)=\int f_{k|k}\left(\left\{x\right\}\cup X_{k}|Z_{k}\right)\delta X_{k}$$

Here, we use the abbreviation $D_{k|k}\left(x\right)=D_{k|k}\left(x|Z_{k}\right)$. In point theory, $D_{k|k}\left(x\right)$ is defined as the intensity density. $D_{k|k}\left(x\right)$ is not a probability density, but it represents the density of expected number of points at x. If S represents a region in the single object state space $X_{0}$, the integral $\int_{S}D_{k|k}\left(x\right)dx$ represents the expected number of targets in the state space S. More information about PHD can be found in [22].

#### 1.2.4. Simulated Tempering

Simulated tempering is a stochastic optimization method that is used for improving the probability of an optimization routine converging to a global optima in highly multimodal objectives [25]. It adopts the properties of thermodynamical systems and deals with two important problems with Markov Chain Monte Carlo (MCMC) methods: one is improving exploration of multimodal distributions, and the other one is allowing estimation of the normalizing constant of the target distribution. Simulated tempering augments the Markov chain state with a discrete index variable controlling the inverse temperature of the system [26].

The objective function that we want to minimize is defined by the energy function ϕ of the system and the variables being optimized with the state x∈X. An increasing schedule of *K* inverse temperatures ${\left\{\beta_{k}\right\}}_{k=1}^{K}$ is chosen with $0=\beta_{1}\leq \beta_{2}\leq \cdots \leq \beta_{K}=1$. The variables x∈X on which the target distribution *P* is defined are augmented with a discrete index variable k∈{0,…,K}. A joint density on the target variable x and temperature index *k* is then defined as

(4) $$p_{x,k}\left(x,k\right)=\frac{1}{C}\exp\left(-\beta_{k}\phi \left(x\right)-\left(1-\beta_{k}\right)\psi \left(x\right)+\omega_{k}\right)$$

where *C* is a constant, and ${\left\{\omega_{k}\right\}}_{k=0}^{K}$ is a set of prior weights associated with each inverse temperature value, and which can be used to help shape the marginal distribution on the temperature index *k*. The energy function ϕ is defined as the negative logarithm of the unnormalized target density i.e., $\phi \left(x\right)=-\log\tilde{p}\left(x\right)$. Function ψ defines a corresponding energy function for a base distribution *Q* with normalized density q(x)=exp(−ψ(x)).

If k=0, the conditional distribution $P_{x|k}$ on the target variables x corresponds to the base distribution *Q* and $\beta_{0}=0$. If k=K, it will correspond to the target distribution *P* and $\beta_{K}=1$. We can therefore use the x components of sampled chain states for which k=K to estimate the variables of interest which is related to the target distribution *P*. In simulated tempering, a Markov chain with invariant distribution corresponding to (4) is constructed by alternating updates of the target variables x given the current value of temperature index *k*, with updates of the temperature index *k* given the current value of the target variables x. More information about simulated tempering can be found in [27].

### 1.3. Motivation and Advantages of the Proposed Parameter Estimation Algorithm

In this paper, we choose to use the Bayesian formulation represented by Equation (2). The unknown parameters are regarded as a random vector and associated with a suitable prior information modeled by the prior distribution. The posterior PDF of the parameters is to be characterized by using the given measurements. The proposed parameter estimation algorithm can be regarded as the Monte Carlo batch techniques [28], and it is perfect for estimating parameters of stochastic dynamic systems. The proposed parameter estimation algorithm is an off-line Bayesian parameter estimation algorithm, and it is an updated version of the marginalization based algorithms.

The conventional Bayesian parameter estimation algorithms depend on the vector based representation of data including states and measurements. The vector based representation makes these algorithms have three essential disadvantages. The first one is that they must assume that the studied system is a single permanently active system and the model is presumed. They cannot be used for estimating the dynamic system that switches on and off randomly. The transition from one mode to another is impossible. The second disadvantage is that they are based on the assumption that the detection is perfect which means no clutters and no missed detections, and they also need the number and sort order of measurements to be previously designated. The third disadvantage is that they are not appropriate for estimating the systems with varying state dimensions caused by the varying number of constituent elements contained in the studied system.

The limits of the commonly used random vector based Bayesian estimation algorithms are analyzed in [29]. We can find that the root of their disadvantages is that they must ensure the dimension and elements’ order in each vector to be equal and fixed. They also need necessary operations outside of the Bayesian recursion to ensure the consistency of the vectors used for calculation. The determination of newly observed measurements and missed measurements is through vector augmentation and truncation which are very computationally intensive and irreversible. In this paper, we propose to employ the RFS theory to overcome these disadvantages of the standard vector based Bayesian parameter estimation algorithms.

An RFS has a broader application prospect than a random vector, because an RFS has a random number of constituent vectors and it allows these constituent vectors themselves to be random, distinct and unordered [30]. In practice, the number of measurements is not fixed and the ordering of measurements is irrelevant, so the measurements are better to be modeled as an RFS. RFS based data representation generalizes the system model and measurement model for parameter estimation, because it takes into account a more realistic situation where the varying number of objects, detection uncertainty, clutters, missed detections and data association uncertainty are all taken into consideration.

Unlike the traditional parameter estimation algorithms and the vector based Bayesian parameter estimation algorithms, the proposed Bayesian parameter estimation algorithm employs RFS based modeling, and has the following advantages:

1. It can estimate the parameters by using the imperfect detections including noises, clutters, data association uncertainty and missed detections. Thus, it enables the measurement model used for parameter estimation to be more universal and consistent with the practical system.
2. It can estimate the parameters for the stochastic systems which consist of a varying number of constituent components or objects. It can take the object birth, object death and spawned objects of the studied system into consideration.
3. The proposed algorithm can also accurately estimate the states of the studied system and the number of objects contained in the studied system while estimating the unknown parameters. The estimation of states and object number depends on the estimated parameters.

The rest of the paper is structured as follows. We present the RFS based modeling for both measurement model and system model in Section 2. A detailed description of the PHD based Bayesian inference and the SMC based implementation are given in Section 3. The proposed RFS based parameter estimation algorithm is detailed in Section 4. After describing the proposed algorithm, in Section 5, we take a particular problem of estimating systematic errors of multiple sensors as an example to verify this algorithm. In order to better promote academic exchanges, we provide the source codes for the experiments in the supplementary materials part. Finally, the conclusion is provided in Section 6.

## 2. RFS Based Modeling

The RFS based parameter estimation algorithm uses two kinds of models to describe the real world: one is the Markov transition density based system model; the other one is the measurement likelihood based measurement model. System model is used to model the state transition of the studied system, and the measurement model is used to model the practical measurement system. Here, we focus on estimating the static parameter vector θ which features in the state space models that represent system dynamics and measurement system using observations from the practical system. The RFS based representation of measurements and states is the foundation of RFS based algorithm. In this section, we give the RFS based problem formulation and the RFS based models used in the proposed parameter estimation algorithm.

### 2.1. Problem Formulation

Suppose at time step k=0,1,2,…, the studied system consists of $n_{k}$ objects. Their states can, respectively, be represented by the vectors $x_{k,1},\ldots ,x_{k,n_{k}}$, and each state vector is obtained from the state space $X\subseteq R^{n_{x}}$. In practice, the number $n_{k}$ and the individual states in X of system’s constituent elements are both time varying and random. In this paper, the system state at *k* is described by an RFS $X_{k}=\left\{x_{k,1},\ldots ,x_{k,n_{k}}\right\}\in F\left(X\right)$. The state evolution of the RFS based system model can be described by the Markov system state process conditioned on the static parameters θ∈Θ. The state evolution is characterized by a prior density $X_{0}∼\mu \left(X_{0}|\theta \right)$ and a multiple objects transition density $\Pi_{k|k-1}\left(X_{k}|X_{k-1},\theta \right)$.

The measurement process is imperfect in the sense that there exists detection uncertainty. The measurement devices usually create false detections. If observation datasets are available at time k=1,2,…, and there are $m_{k}$ elements in the observation dataset at time *k*, and the observations take a values in the measurement space $Z\subseteq R^{n_{z}}$. Then, the observations can be modeled by an RFS $Z_{k}=\left\{z_{k,1},\ldots ,z_{k,m_{k}}\right\}\in F\left(Z\right)$. Here, we assume that the measurement process is conditionally independent from the system’s state evolution, and can be fully described by the likelihood $\varphi_{k}\left(Z_{k}|X_{k},\theta \right)$ which is conditioned on θ∈Θ.

We assign a prior distribution to the unknown parameter vector θ, and estimate it on the parameter vector space according to Bayesian theory. The parameter estimation problem in this paper mainly refers to estimating the posterior PDF $p(\theta |Z_{1:K})$; here, $Z_{1:K}=Z_{1},\ldots ,Z_{K}$ is the measurement set sequence gathered up to time index *K*. If the prior density $p_{0}\left(\theta \right)$ is given, according to Equation (2), the estimation result in the Bayesian approach is given by:

(5) $$p\left(\theta |Z_{1:K}\right)=\frac{f\left(Z_{1:K}|\theta \right)p_{0}\left(\theta \right)}{f(Z_{1:K})}\propto f\left(Z_{1:K}|\theta \right)p_{0}\left(\theta \right)$$

The computation of $f(Z_{1:K}|\theta )$ is quite difficult, because it refers to performing estimation on the joint space of the unknown system states history $X_{1:K}$ and the unknown parameter vector θ. To solve this problem, we choose to consider a broader problem instead of solving the problem directly. The parameter estimation problem can be regarded as a subproblem of the chosen problem where the main task is to obtain the following posterior PDF:

(6) $$f\left(\theta ,X_{1:K}|Z_{1:K}\right)\propto f\left(X_{1:K}|Z_{1:K},\theta \right)f\left(Z_{1:K}|\theta \right)p_{0}\left(\theta \right)$$

where $f(X_{1:K}|Z_{1:K},\theta )$ is the posterior PDF of the system states conditioned on θ and all the given observation datasets.

### 2.2. RFS Based Measurement Model

For the given predicted system state $X_{k}=\left\{x_{1},\ldots ,x_{n}\right\}$, the random measurement set collected by the sensor can be represented by $Z_{k}=\Gamma_{k}\left(X_{k}\right)\cup C_{k}$, where $\Gamma_{k}\left(X_{k}\right)$ is the object detection set and it has the form $\Gamma_{k}\left(X_{k}\right)=\Gamma_{k}\left(x_{1}\right)\cup \cdots \cup \Gamma_{k}\left(x_{n}\right)$; $\Gamma_{k}\left(x_{j}\right)$ is the detection set for state $x_{j}$; and $C_{k}$ is the set of Poisson clutters.

Any given object in the studied system can generate either a single measurement or no measurement at all. Consequently, $\Gamma_{k}\left(x_{i}\right)$ can be modeled as $\Gamma_{k}\left(x_{i}\right)=\emptyset^{p_{D}\left(x_{i}|\theta \right)}\cap \left\{Z_{i}\right\}$. Here, $Z_{i}=h\left(x_{i},W_{i}|\theta \right)$ is the sensor-noise model associated with *i*th state $x_{i}$; and $\emptyset^{p_{D}\left(x_{i}|\theta \right)}$ is the discrete random subset of the baseline measurement space $Z_{0}$. Detection uncertainty is modeled as a Bernoulli RFS as follows:

(7) $$Pr\left(\emptyset^{p}=T\right)=\left\{\begin{array}{cc} 1-p_{D}\left(x|\theta \right), & \mathrm{if}\;\;T=\emptyset \\ p_{D}\left(x|\theta \right)\cdot g\left(Z_{i}|\theta \right), & \mathrm{if}\;\;T=Z_{0}\\ 0, & \mathrm{otherwise} \end{array}\right.$$

where $p_{D}\left(x|\theta \right)$ is the probability that a single object whose state is x gives a single observation, and $1-p_{D}\left(x|\theta \right)$ is the probability that the sensor generates no measurement at all.

The fact that the clutter process is Poisson means that the clutters can be represented by the set $C=\{c_{1},\ldots ,c_{M}\}$, where *M* is a random nonnegative integer with probability distribution $p_{M}\left(m\right)=e^{-\lambda }\lambda^{m}/m!$; $c_{1},\ldots ,c_{M}$ are independent, and identically distributed random measurement vectors with density $c\left(z\right)=g_{C}\left(z|\theta \right)$.

For the state set $X_{k}$, the PDF of receiving the measurement set $Z_{k}$ is described by the true likelihood function $\varphi_{k}\left(Z_{k}|X_{k},\theta \right)$, which describes the sensor measurement for the studied system and characterizes the missed detections, clutters, and object generated observations. As described in [29], the true likelihood function can be calculated as follows:

(8) $$\begin{array}{cc} \varphi_{k}\left(Z_{k}|X_{k},\theta \right) & =\varphi_{k}\left(\left(\Gamma_{k}\left(X_{k}\right)\cup C_{k}\right)|X_{k},\theta \right)=\sum_{W\subseteq Z_{k}}p_{\Gamma_{k}\left(X_{k}\right)}\left(W|X_{k},\theta \right)p_{C_{k}|\theta }\left(Z_{k}-W\right) \end{array}$$

(9) $$\begin{array}{c} =e^{\lambda }\cdot g_{C}\left(Z_{k}|\theta \right)\cdot g_{k}\left(\emptyset |X_{k},\theta \right)\cdot \sum_{\xi }\prod_{i:\xi (i)>0}\frac{p_{D}\left(x_{i}|\theta \right)\cdot g_{k}\left(z_{\xi (i)}|x_{i},\theta \right)}{\left(1-p_{D}\left(x_{i}|\theta \right)\right)\cdot \lambda \cdot c\left(z_{\xi (i)}|\theta \right)} \end{array}$$

where $g_{C}\left(Z_{k}|\theta \right)=e^{-\lambda }\prod_{z\in Z_{k}}\lambda c\left(z|\theta \right)$ and $g_{k}\left(\emptyset |X_{k},\theta \right)=e^{-\lambda }\prod_{x\in X_{k}}\left(1-p_{D}\left(x|\theta \right)\right)$; the summation is taken over all associated hypothesis ξ:{1,…,n}⟶{0,1,…,m}. In time, the clutter process is Poisson distributed, and the mean value is λ. In space, the clutter process is distributed according to an arbitrary density c(z), and $c_{k|k}\left(Z_{k}|\theta \right)=e^{-\lambda }\lambda_{k|k}^{m}c_{k|k}\left(z_{1}\right)\cdots c_{k|k}\left(z_{m}\right)$ is the probability that a set $Z_{k}=\left\{z_{1},\ldots ,z_{m}\right\}$ of clutters will be generated. $g_{k+1}\left(z|x,\theta \right)=g_{W_{k+1}}\left(z-h_{k+1}\left(x|\theta \right)\right)$ is the likelihood function of a single sensor.

### 2.3. RFS Based System Model

Some systems may consist of multiple objects, and the number of these objects can be constantly varying, just as the objects appear and disappear from the system. Existing objects can give rise to new objects through spawning. Objects can likewise leave the system, as when disappearing, or they can be damaged or destroyed. All of these instances of multiple object dynamics should be explicitly modeled. If multiple objects contained in the studied system are related to birth, death, spawning, merging, and so on, the standard vector based estimation algorithms are inapplicable, because they fail to accurately estimate the number of objects in the studied system.

We model the time evolution of the system which consists of multiple objects by employing RFS. We take the object birth, death, spawn, and motion into consideration. The system Markov density $\Pi_{k|k-1}\left(X_{k}|X_{k-1},\theta \right)$ characterizes the time evolution of the system states. Here, we give the true system Markov density function $\Pi_{k|k-1}\left(X_{k}|X_{k-1},\theta \right)$ for the proposed system model, where $X_{k}=\left\{x_{1},\ldots ,x_{n_{k}}\right\}$ and $X_{k-1}=\left\{x_{1}^{\prime },\ldots ,x_{n_{k-1}}^{\prime }\right\}$. The RFS based representation of system’ states has the following mathematical form

(10) $$X_{k}=S_{k|k-1}\left(X_{k-1}\right)\cup B_{k|k-1}\left(X_{k-1}\right)\cup B_{k}$$

where $X_{k}$ is the set of predicted system states, $S_{k|k-1}\left(X_{k-1}\right)$ is a set of states of persisting objects that are continuing to exist in the system, $B_{k|k-1}\left(X_{k-1}\right)$ represents the state set of spawned elements, $B_{k}$ represents the state set of spontaneous objects. $S_{k|k-1}\left(X_{k-1}\right)$ has the form $S_{k|k-1}\left(X_{k-1}\right)=S_{k|k-1}\left(x_{1}^{\prime }\right)\cup \cdots \cup S_{k|k-1}\left(x_{n_{k-1}}^{\prime }\right)$, and $S_{k|k-1}\left(x_{i}^{\prime }\right)$ is the set of predicted states evolved from $x_{i}^{\prime }$. $B_{k|k-1}\left(X_{k-1}\right)$ has the form $B_{k|k-1}\left(X_{k-1}\right)=B_{k|k-1}\left(x_{1}^{\prime }\right)\cup \cdots \cup B_{k|k-1}\left(x_{n_{k-1}}\right)$, and $B_{k|k-1}\left(x_{i}^{\prime }\right)$ is the set of spawned states by the object whose previous state is $x_{i}^{\prime }$.

As detailed in [29], the RFS based system model is identical in mathematical form to the RFS based measurement model. The corresponding true Markov density is

(11) $$\begin{array}{c} \Pi_{k|k-1}\left(X_{k}|X_{k-1},\theta \right)=\Pi_{k|k-1}\left(\left(S_{k|k-1}\cup B_{k|k-1}\cup B_{k}\right)|X_{k-1},\theta \right) \end{array}$$

(12) $$\begin{array}{c} =\sum_{W_{1}\cap W_{2}=\emptyset ,W_{1}\cup W_{2}\subseteq X_{k-1}}p_{S_{k|k-1}}\left(W_{1}|X_{k-1},\theta \right)p_{B_{k|k-1}}(W_{2}|X_{k-1}\left|\theta \right)\cdot p_{B_{k}}\left(X_{k}-W_{1}-W_{2}|\theta \right) \end{array}$$

(13) $$\begin{array}{c} =e^{\mu (X_{k-1}|\theta )}f_{B_{k|k-1}}\left(X_{k}|\theta \right)f_{k|k-1}\left(\emptyset |X_{k-1},\theta \right)\sum_{\xi }\prod_{\xi (i)>0}\frac{p_{s}\left(x_{i}^{\prime }|\theta \right)\pi_{k|k-1}\left(x_{\xi (i)}|x_{i}^{\prime },\theta \right)}{\left(1-p_{s}\left(x_{i}^{\prime }|\theta \right)\right)\mu \left(X_{k-1}\right)b\left(x_{\xi (i)}|X_{k-1},\theta \right)} \end{array}$$

where $\pi_{k|k-1}\left(x_{k}|x_{k-1},\theta \right)$ is a Markov transition density, which corresponds to a single object system model $x_{k}=\pi \left(x_{k-1},V_{k-1}|\theta \right)$. It is the likelihood that a single object will have state vector $x_{k}$ at time step *k* if it had state vector $x_{k-1}$ at time step k−1. $p_{s}\left(x_{k}|\theta \right)\overset{abbr.}{=}p_{s}^{k|k-1}\left(x_{k-1}|\theta \right)$ denotes the probability of object’s surviving into time step *k*. $b(B_{k|k-1}\left(X_{k-1}\right)|x_{k-1},\theta )$ is the probabilistic distribution of spawning a set $B_{k|k-1}\left(X_{k-1}\right)$ of new objects at time step *k*. The summation is taken over all association hypotheses $\xi :\left\{1,\ldots ,n_{k-1}\right\}⟶\left\{0,1,\ldots ,n_{k}\right\}$.

The related definitions in Equation (13) are as follows:

(14) $$f_{k|k-1}\left(\emptyset |X_{k-1},\theta \right)=e^{-\mu (X_{k-1}|\theta )}\prod_{x_{k-1}\in X_{k-1}}\left(1-p_{s}\left(x_{k-1}|\theta \right)\right)$$

(15) $$\mu \left(X_{k-1}|\theta \right)=\mu_{0}+\mu_{1}\left(x^{\prime }|\theta \right)+\cdots +\mu_{n_{k-1}}\left(x^{\prime }|\theta \right)$$

(16) $$b\left(x|X_{k-1},\theta \right)=\frac{\mu_{0}b_{0}\left(x|\theta \right)+\mu_{1}\left(x^{\prime }|\theta \right)b\left(x|x_{1}^{\prime },\theta \right)+\cdots +\mu_{n_{k-1}}\left(x^{\prime }|\theta \right)b\left(x|x_{n_{k-1}}^{\prime },\theta \right)}{\mu_{0}+\mu_{1}\left(x^{\prime }|\theta \right)+\cdots +\mu_{n_{k-1}}\left(x^{\prime }|\theta \right)}$$

Here, $\mu_{0}$ is the expected number of spontaneously generated new objects and $b_{0}\left(x|\theta \right)$ is their physical distribution. In addition, $\mu (x_{i}^{\prime }|\theta )$ is the expected number of new objects spawned by an object whose previous state is $x_{i}^{\prime }$, and $b(x|x_{i}^{\prime },\theta )$ is their physical distribution.

## 3. PHD Filter

### 3.1. PHD Based Bayesian Equations

The Bayesian estimator sequentially determines the posterior PDF of the system states $f_{k|k}=\left(X_{k}|Z_{1:k},\theta \right)$ at each time step *k*, where $Z_{1:k}\equiv Z_{1},\ldots ,Z_{k}$ denotes the gathered measurement set sequence up to time step *k*. The posterior PDF is calculated through the prediction and the correction recursion sequentially. On the assumption that the posterior PDF $f_{k-1|k-1}\left(X_{k-1}|Z_{1:k-1},\theta \right)$ at time k−1 is already known, if the set of measurements $Z_{k}$ which is related to time *k* has been given, the prediction step and corrected step can be described as in [22]:

(17) $$f_{k|k-1}\left(X_{k}|Z_{1:k-1},\theta \right)=\int \Pi_{k|k-1}\left(X_{k}|X_{k-1},\theta \right)\cdot f_{k-1|k-1}\left(X_{k-1}|Z_{1:k-1},\theta \right)\delta X_{k-1}$$

(18) $$f_{k|k}\left(X_{k}|Z_{1:k},\theta \right)=\frac{\varphi \left(Z_{k}|X_{k},\theta \right)\cdot f_{k|k-1}\left(X_{k}|Z_{1:k-1},\theta \right)}{\int \varphi \left(Z_{k}|X,\theta \right)\cdot f_{k|k-1}\left(X|Z_{1:k-1},\theta \right)\delta X}$$

The PHD filter is an approximation of the Bayesian estimator described by Equations (17) and (18) [31]. Rather than propagating the FISST PDF $f_{k|k}\left(X_{k}|Z_{1:k},\theta \right)$, we can only propagate PHD $D_{k|k}\left(x|Z_{1:k},\theta \right)$, defined by Equation (3).

The prediction equation of the PHD filter can be represented as follows [21]:

(19) $$D_{k|k-1}\left(x|\theta \right)=\gamma_{k|k-1}\left(x|\theta \right)+p_{s}\int \pi_{k|k-1}\left(x|x^{\prime },\theta \right)\cdot D_{k-1|k-1}\left(x^{\prime }|\theta \right)dx^{\prime }$$

where $\gamma_{k|k-1}\left(x|\theta \right)$ represents the PHD related to the object birth RFS between step k−1 and *k*. Once the observation dataset $Z_{k}$ for time *k* is available, the correction equation of the PHD filter can be represented as follows [21]:

(20) $$D_{k|k}\left(x|\theta \right)=\left(1-p_{D}\right)\cdot D_{k|k-1}\left(x|\theta \right)+\sum_{z\in Z_{k}}\frac{p_{D}\cdot g_{k}\left(z|x,\theta \right)\cdot D_{k|k-1}\left(x|\theta \right)}{\kappa_{k}\left(z|\theta \right)+p_{D}\int g_{k}\left(z|x,\theta \right)\cdot D_{k|k-1}\left(x|\theta \right)dx}$$

where $\kappa_{k}\left(z|\theta \right)$ is the PHD related to the clutter RFS at step *k*. Here, the clutters are characterized by the Poisson RFS. The PHD of the Poisson RFS is $\kappa_{k}\left(z|\theta \right)=\lambda c\left(z|\theta \right)$; here, λ denotes the mean number at step *k*, and c(z|θ) denotes the spatial distribution of clutters in the measurement space.

### 3.2. SMC based Implementation

Beginning with Sidenbladh [32] and Zajic [33], many researchers have studied how to implement the PHD filter by using the SMC method. By using sequential importance sampling to each terms of Equation (19), the particle approximation of Equation (19) can be obtained. After choosing the importance densities $p_{k}\left(\cdot |Z_{k},\theta \right)$ and $q_{k}\left(\cdot |x_{k-1},Z_{k},\theta \right)$ for spontaneous birth and persisting objects, Equation (19) can be rewritten as

(21) $$D_{k|k-1}\left(x_{k}|\theta \right)=\frac{\gamma_{k|k-1}\left(x_{k}|\theta \right)}{p_{k}\left(x_{k}|Z_{k},\theta \right)}p_{k}\left(x_{k}|Z_{k},\theta \right)+\sum_{i=1}^{L_{k-1}}\omega_{k-1}^{\left(i\right)}\frac{p_{s}\cdot \pi_{k|k-1}\left(x_{k}|x_{k-1}^{\left(i\right)},\theta \right)}{q_{k}\left(x_{k}|x_{k-1}^{\left(i\right)},Z_{k},\theta \right)}q_{k}\left(x_{k}|x_{k-1}^{\left(i\right)},Z_{k},\theta \right)$$

Therefore, we can get the particle approximation of $D_{k|k-1}\left(x|\theta \right)$ as follows

(22) $$D_{k|k-1}\left(x|\theta \right)=\sum_{i=1}^{L_{k-1}+J_{k}}\omega_{k|k-1}^{\left(i\right)}\delta_{x_{k|k-1}^{\left(i\right)}}\left(x\right)$$

where

(23) $$x_{k|k-1}^{\left(i\right)}∼\left\{\begin{array}{c} q_{k}\left(\cdot |x_{k-1}^{\left(i\right)},Z_{k},\theta \right),i=1,\ldots ,L_{k-1}\\ p_{k}\left(\cdot |Z_{k},\theta \right),i=L_{k-1}+1,\ldots ,L_{k-1}+J_{k} \end{array}\right.$$

(24) $$\omega_{k|k-1}^{\left(i\right)}=\left\{\begin{array}{c} \frac{p_{s}\pi_{k|k-1}\left(x_{k|k-1}^{\left(i\right)}|x_{k-1}^{\left(i\right)},\theta \right)}{q_{k}\left(x_{k|k-1}^{\left(i\right)}|x_{k-1}^{\left(i\right)},Z_{k},\theta \right)}\omega_{k-1}^{\left(i\right)},i=1,\ldots ,L_{k-1}\\ \frac{\gamma_{k|k-1}\left(x_{k|k-1}^{\left(i\right)}|\theta \right)}{J_{k}p_{k}\left(x_{k|k-1}^{\left(i\right)}|Z_{k},\theta \right)},i=L_{k-1}+1,\ldots ,L_{k-1}+J_{k} \end{array}\right.$$

For the correction step of the PHD filter, the predicted PHD can be represented by ${\left\{\omega_{k|k-1}^{\left(i\right)},x_{k}^{\left(i\right)}\right\}}_{i=1}^{L_{k-1}+J_{k}}$ after prediction step, where $x_{k}^{\left(i\right)}=x_{k|k-1}^{\left(i\right)}$. Applying the correction Equation (20), we can get the following particle approximation

(25) $$D_{k|k}\left(x|\theta \right)=\sum_{i=1}^{L_{k-1}+J_{k}}\omega_{k}^{\left(i\right)}\delta_{x_{k}^{\left(i\right)}}\left(x\right)$$

where

(26) $$\omega_{k}^{\left(i\right)}=\left(1-p_{D}\right)\omega_{k|k-1}^{\left(i\right)}+\sum_{z\in Z_{k}}\frac{p_{D}\cdot g_{k}\left(z|x_{k}^{\left(i\right)},\theta \right)\cdot \omega_{k|k-1}^{\left(i\right)}}{\kappa_{k}\left(z|\theta \right)+\sum_{j=1}^{L_{k-1}+J_{k}}p_{D}\cdot g_{k}\left(z|x_{k}^{\left(j\right)},\theta \right)\cdot \omega_{k|k-1}^{\left(j\right)}}$$

By updating the weights of the particles in term of Equation (26), the correction step maps the predicted particle system ${\left\{\omega_{k|k-1}^{\left(i\right)},x_{k}^{\left(i\right)}\right\}}_{i=1}^{L_{k-1}+J_{k}}$ into the corrected particle system ${\left\{\omega_{k}^{\left(i\right)},x_{k}^{\left(i\right)}\right\}}_{i=1}^{L_{k-1}+J_{k}}$.

## 4. RFS based Parameter Estimation Algorithm

The system states conditioned on θ, are estimated by using the Bayesian theory. The main task is to obtain an estimate of PDF $f(X_{1:K}|Z_{1:K},\theta )$ for each possible value of θ by running a PHD filter implemented by SMC. By this way, we can evaluate the measurement likelihood $f(Z_{1:K}|\theta )$, and then we can indirectly obtain a practical solution for $p(\theta |Z_{1:K})$.

### 4.1. Formula Derivation

By employing PHD to approximate Equation (6), we can estimate $f(X_{1:K}|Z_{1:K},\theta )$ which depends on RFS based modeling. According to the relationship between the PHD and the RFS based PDF detailed in [22], Equation (6) can be rewritten as the following equation

(27) $$D\left(\theta ,x_{1:K}|Z_{1:K}\right)\propto D\left(x_{1:K}|Z_{1:K},\theta \right)\cdot f\left(Z_{1:K}|\theta \right)\cdot p_{0}\left(\theta \right)$$

The advantage of Equation (27) is that the posterior PHD $D(x_{1:K}|Z_{1:K},\theta )$ is defined on the standard vector based state space X, so the calculation of the RFS based posterior PDF $f(X_{1:K}|Z_{1:K},\theta )$ is quite easy.

The observed data likelihood $f(Z_{1:K}|\theta )$ of the accumulated sequence of observation dataset which features in Equations (5) and (27) can be calculated by using the following equation:

(28) $$f\left(Z_{1:K}|\theta \right)=\prod_{k=1}^{K}f\left(Z_{k}|Z_{1:k-1},\theta \right)$$

Now, the problem is how to calculate the measurement likelihood $f(Z_{k}|Z_{1:k-1},\theta )$ using the results of PHD filter. The equations and SMC based implementation of the PHD filter for recursively estimating $D_{k|k}\left(x|Z_{1:k},\theta \right)$ have been introduced in Section 3.1 and Section 3.2. The measurement likelihood $f(Z_{k}|Z_{1:k-1},\theta )$, which is defined as in [21], can be calculated as follows

(29) $$f\left(Z_{k}|Z_{1:k-1},\theta \right)=\int \varphi \left(Z_{k}|X_{k},\theta \right)\cdot \Pi \left(X_{k}|Z_{1:k-1},\theta \right)\delta X_{k}$$

From Equations (28) and (29), we can find the tight relationship between the parameter estimation problem and the state estimation problem. The key challenge is how to deal with the unknown system states. The PHD filter implemented by the SMC method is used to solve this problem. Thus, $f(Z_{k}|Z_{1:k-1},\theta )$ can be obtained by using PHD filer, and can be calculated as follows:

(30) $$\begin{array}{cc} f(Z_{k}|Z_{1:k-1},\theta ) & =\alpha e^{-\int p_{D}\left(x_{k}|\theta \right)\cdot D_{k|k-1}\left(x_{k}|Z_{1:k-1},\theta \right)dx_{k}}\\ & \times \prod_{z\in Z_{k}}\left(\kappa_{k}\left(z|\theta \right)+\int p_{D}\left(x_{k}|\theta \right)\cdot g\left(z|x_{k},\theta \right)\cdot D_{k|k-1}\left(x_{k}|Z_{1:k-1},\theta \right)dx_{k}\right) \end{array}$$

where α is a proportionality constant.

As described in Section 3.2, we can use the weighted particle system ${\left\{x_{k}^{\left(i\right)},\omega_{k}^{\left(i\right)}\right\}}_{i=1}^{N}$ to represent the posterior PHD $D_{k|k}\left(x|Z_{1:k},\theta \right)$. To estimate the posterior $p(\theta |Z_{1:K})$, we should firstly obtain the approximation of $f(Z_{k}|Z_{1:k-1},\theta )$ given by Equation (30) by running the PHD filter. According to Equation (22), the predicted PHD $D_{k|k-1}\left(x|Z_{1:k-1},\theta \right)$ is approximated by the following particles

(31) $${\hat{D}}_{k|k-1}\left(x|Z_{1:k-1},\theta \right)=\sum_{i=1}^{N_{k}}\omega_{k|k-1}^{\left(i\right)}\delta_{x_{k|k-1}^{\left(i\right)}}\left(x\right)$$

Substituting Equation (31) into Equation (30), we can obtain the following result:

(32) $$\begin{array}{cc} \hat{f}\left(Z_{k}|Z_{1:k-1},\theta \right) & =\alpha \exp\left\{-\sum_{i=1}^{N_{k}}p_{D}\left(x_{k|k-1}^{\left(i\right)}|\theta \right)\omega_{k|k-1}^{\left(i\right)}\right\}\\ & \times \prod_{z\in Z_{k}}\left(\kappa_{k}\left(z|\theta \right)+\sum_{i=1}^{N_{k}}p_{D}\left(x_{k|k-1}^{\left(i\right)}|\theta \right)\cdot g\left(z|x_{k|k-1}^{\left(i\right)},\theta \right)\omega_{k|k-1}^{\left(i\right)}\right) \end{array}$$

Therefore, according to Equation (28), $f(Z_{1:K}|\theta )$ can be calculated as follows:

(33) $$\begin{array}{cc} \hat{f}\left(Z_{1:K}|\theta \right) & =\alpha^{K}\exp\left\{-\sum_{k=1}^{K}\sum_{j=1}^{N_{k}}p_{D}\left(x_{k|k-1}^{\left(j\right)}|\theta \right)\omega_{k|k-1}^{\left(j\right)}\right\}\\ & \times \prod_{k=1}^{K}\prod_{z\in Z_{k}}\left(\kappa_{k}\left(z|\theta \right)+\sum_{j=1}^{N_{k}}p_{D}\left(x_{k|k-1}^{\left(j\right)}|\theta \right)\cdot g\left(z|x_{k|k-1}^{\left(j\right)},\theta \right)\omega_{k|k-1}^{\left(j\right)}\right) \end{array}$$

### 4.2. Simulated Tempering Based Importance Sampling

We have obtained the estimate of $f(Z_{1:K}|\theta )$ by running the PHD filter, the following step is to compute $p(\theta |Z_{1:K})$ according to Equation (5). Since the analytic solution for $p(\theta |Z_{1:K})$ is difficult to compute, we solve Equation (5) by using the simulated tempering based importance sampling method.

Since we are interested in the values of the unknown parameters, we can compute them as the posterior mean by using the following equation:

(34) $$E\left(\theta |Z_{1:K}\right)=\int \theta p\left(\theta |Z_{1:K}\right)d\theta$$

To avoid doing integral calculus, we propose to approximate Equation (34) via importance sampling. Here, we assume the sample size is *M*, we draw a sample $\theta_{1},\ldots ,\theta_{M}$ from the importance density ξ. Thus, the integral in Equation (34) can be approximated by the following equation:

(35) $$\hat{\theta }=\sum_{j=1}^{M}\ell_{j}\theta_{j}$$

The sample weights $\ell_{j}\geq 0,\left(j=1,\ldots ,M\right)$ are defined as:

(36) $$\ell_{j}=\frac{{\tilde{\ell }}_{j}}{\sum_{m=1}^{M}{\tilde{\ell }}_{m}},\;\mathrm{with}\;\;{\tilde{\ell }}_{j}=\frac{f\left(Z_{1:K}|\theta_{j}\right)p_{0}\left(\theta_{j}\right)}{\xi (\theta_{j})}$$

According to Equation (36), we find that α can be omitted, since it cancels out if we normalize the weights. Thus, for convenience, we assume α=1. The determination of sample weight $\ell_{j}$ depends on the selection of importance density $\xi (\theta_{j})$. A good selection of importance density should be proportional to the posterior PDF $f(Z_{1:K}|\theta_{j})$ and produce sample weights with a small variance.

Since we can only obtain an estimation of $f\left(Z_{1:K}|\theta_{j}\right)/\alpha^{K}$ after running the SMC based PHD filter, the selection of the importance density ξ is quite important. If the sample size M→∞, the approximation Equation (35) will be quite accurate for many importance densities. However, if *M* is finite, the approximation accuracy will depend greatly on the specific selection of importance density ξ. As shown in Equation (36), the accuracy of importance sampling by using the prior information is not satisfactory for any possible sample size, since the posterior PDF $p(\theta |Z_{1:K})$ which is proportional to $f\left(Z_{1:K}|\theta \right)p_{0}\left(\theta \right)$ is unknown. We propose a simulated tempering [2] based importance sampling method to obtain a better importance density ξ which is similar to the posterior PDF $p(\theta |Z_{1:K})$.

Here, we use *S* to represent the maximum stages number and $\vartheta_{s}$, s=1,…,S, represent the proposal density for the *s*-th stage. The main idea of tempering is to sequentially generate a serious of importance densities from which we can sequentially draw samples. The first importance density usually resembles the prior density $p_{0}\left(\theta \right)$, and the final importance density is the posterior PDF $p(\theta |Z_{1:K})$. The sequential importance density in the sequence should have very small difference. We obtain $\vartheta_{S}=p\left(\theta |Z_{1:K}\right)$ at the final stage *S* . A sequence of importance densities that begin with the prior density and increasingly resemble the posterior PDF can be generated by the following equation, for s=1,…,S

(37) $$\vartheta_{s}\left(\theta \right)\propto {\left[f\left(Z_{1:K}|\theta \right)\right]}^{\Lambda_{s}}p_{0}\left(\theta \right)$$

where $\Lambda_{s}=\sum_{j=1}^{s}\beta_{j}$ with $\beta_{j}\in \left(0,1\right]$ and at the final stage we have $\Lambda_{S}=1$. Thus, $\Lambda_{s}$ increases with the growth of *s* and its upper bound is 1.

During the tempering process, we should sequentially draw samples from importance densities $\vartheta_{1},\vartheta_{2},\ldots ,\vartheta_{S}$. At the first stage (s=1), we draw the sample ${\left\{\theta_{j}^{1}\right\}}_{j=1}^{M}$ from the prior density $\vartheta_{0}\left(\theta \right)=p_{0}\left(\theta \right)$. At the *s*-th stage, we use the sample ${\left\{\theta_{j}^{s-1}\right\}}_{j=1}^{M}$ drawn from $\vartheta_{s-1}$ at the (s−1)th stage to obtain a sample from $\vartheta_{s}$. Firstly, we need to compute weights from particles in ${\left\{\theta_{j}^{s-1}\right\}}_{j=1}^{M}$ by using the equation ${\tilde{\ell }}_{j}^{s}=f{\left(Z_{1:K}|\theta_{j}^{s-1}\right)}^{\beta_{s}}$ for j=1,…,M. Then, we normalize the weights.

Resampling is quite necessary for tempering, because it can help to remove the particles with lower weights from the sample ${\left\{\theta_{j}^{s-1}\right\}}_{j=1}^{M}$ and improve the diversity of the remaining particles. Thus, the second step is resampling. Resampling means selecting *M* indices $i_{1}^{s},\ldots ,i_{M}^{s}$ with probability $Pr\left\{i_{j}^{s}=j\right\}=\ell_{j}^{s}$.

After resampling, the particles ${\left\{{\tilde{\theta }}_{j}^{s}\right\}}_{1\leq j\leq M}$ will almost surely have repeated elements, because the weights $\ell_{1}^{s},\ldots ,\ell_{M}^{s}$ will be the most possible to be uneven. Here, the MCMC (Markov Chain Monte Carlo) [34] is employed to the sample ${\left\{{\tilde{\theta }}_{j}^{s}\right\}}_{1\leq j\leq M}$ that have been resampled. MCMC can help to remove the duplicate particles, thus the diversity of the sample will be increased. For each particle of ${\left\{{\tilde{\theta }}_{j}^{s}\right\}}_{1\leq j\leq M}$, a new sample particle can be drawn as follows

(38) $$\theta_{j}^{s,*}∼g_{s}\left(\cdot |{\tilde{\theta }}_{j}^{s}\right)$$

where $g_{s}$ represents the importance density for the *s*-th stage. The decision of accepting or rejecting $\theta_{j}^{s,*}$ with certain probability is made by using the Metropolis–Hastings algorithm. If the MCMC move is accepted, $\theta_{j}^{s}=\theta_{j}^{s,*}$. If the move is rejected, $\theta_{j}^{s}={\tilde{\theta }}_{j}^{s}$. Thus, we can obtain a new sample ${\left\{\theta_{j}^{s}\right\}}_{j=1}^{M}$. The probability of accepting the MCMC move is determined by the following equation

(39) $$\eta =\min\left\{1,\frac{\vartheta_{s}\left(\theta_{j}^{s,*}\right)g_{s}\left({\tilde{\theta }}_{j}^{s}|\theta_{j}^{s,*}\right)}{\vartheta_{s}\left({\tilde{\theta }}_{j}^{s}\right)g_{s}\left(\theta_{j}^{s,*}|{\tilde{\theta }}_{j}^{s}\right)}\right\}$$

To diversify the sample, the importance density $g_{s}$ should generate the candidate particles over a fairly large area of the parameter space. We should ensure that $g_{s}$ is still within the area where the likelihood is high. We can select the importance density by the following method as introduced in [35]:

(40) $$g_{s}\left(\theta^{*}|\theta \right)=g_{s}\left(\theta^{*}\right)=N\left(\theta^{*};{\hat{\mu }}_{s},{\hat{C}}_{s}\right)$$

where N(θ;μ,C) denotes a Gaussian distribution; ${\hat{\mu }}_{s}$ and ${\hat{C}}_{s}$ are respectively the mean and covariance matrix of the weighted particles ${\left\{\ell_{j}^{s},\theta_{j}^{s-1}\right\}}_{j=1}^{M}$.

The pseudo-code of the proposed parameter estimation algorithm is shown in Algorithm 1. Here, 1≤H≤M and ϕ>0 are parameters that should be defined by users.

**Algorithm 1:** Random Finite Set based Parameter Estimation Algorithm

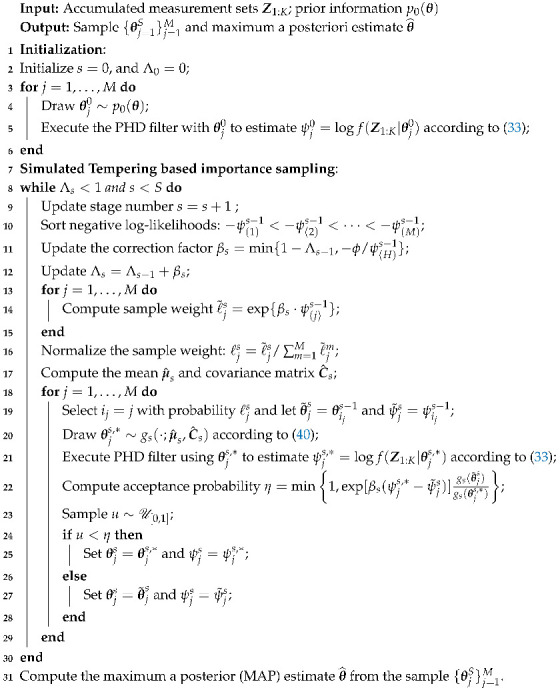



## 5. Experiments

In this section, we verify the correctness and validity of the proposed algorithm by using an example of multiple sensors. Estimating the systematic errors of multiple sensors is important for sensor registration in cooperative networked surveillance [36]. In this example, we estimate the systematic errors of two sensors. The advantages of the proposed algorithm introduced in Section 1.3 are all successfully proved. In order to ensure that those who are interested in the experiments can conduct these experiments smoothly, we also provide the source codes as the supplementary materials.

### 5.1. Experimental Models

Here, we use the measurements given by *R* (here R=2) static sensors to estimate the systematic errors of these sensors. Here, we denote the observation dataset reported by sensor $r_{k}\in \left\{1,\ldots ,R\right\}$ at time *k* by $Z_{k}^{\left(r_{k}\right)}=\left\{z_{k,1}^{\left(r_{k}\right)},\ldots ,z_{k,m_{k,r_{k}}}^{\left(r_{k}\right)}\right\}$. To verify the first advantage introduced in Section 1.3, we use the detection probability $p_{D}$ to characterize the detection uncertainty, and missed detection. The sensor observations are affected by the systematic errors and the measurement noises. The clutters are characterized by using Poisson RFS. We assume that the observations provided by the sensors are the azimuth and range to the objects in the surveillance system. For an observation $z\in Z_{k}^{\left(r_{k}\right)}$ which is related to the object with state $x\in X_{k}$, the measurement likelihood of the *r*-th sensor is modeled as:

(41) $$g_{k}^{\left(r_{k}\right)}\left(z|x,\theta \right)=N\left(z;h_{k}^{\left(r_{k}\right)}\left(x\right)-\theta^{\left(r_{k}\right)},\Sigma_{k}^{\left(r_{k}\right)}\right)$$

where $h_{k}^{\left(r_{k}\right)}\left(x\right)$ is the measurement function of the *r*-th sensor, and its specific form is as follows:

(42) $$h_{k}^{\left(r_{k}\right)}\left(x\right)=\left[\begin{array}{c} \sqrt{{\left(x-x_{r}\right)}^{2}+{\left(y-y_{r}\right)}^{2}}\\ \arctan(\frac{x-x_{r}}{y-y_{r}}) \end{array}\right]$$

where $\left(x_{r},\;y_{r}\right)$ is the position of the *r*-th sensor. The unknown parameter vector θ consists of four parameters as follows:

(43) $$\theta ={\left[\Delta \eta_{1},\;\Delta \rho_{1},\;\Delta \eta_{2},\;\Delta \rho_{2}\right]}^{T}$$

where $\theta^{\left(r\right)}={\left[\Delta \eta_{r},\;\Delta \rho_{r}\right]}^{T}$, which appears in Equation (41), denotes the systematic error vector for the *r*-th sensor (r=1,2), Δη is the azimuth systematic error and Δρ is the range systematic error. $\Sigma_{k}^{\left(r_{k}\right)}=diag\left[{\left(\sigma_{\eta }^{\left(r\right)}\right)}^{2},{\left(\sigma_{\rho }^{\left(r\right)}\right)}^{2}\right]$ is the covariance matrix in (41). $p_{D}^{\left(r\right)}$ denotes the detection probability of the *r*-th sensor.

To verify the second advantage of the proposed algorithm introduced in Section 1.3, the targeted system surveilled by the sensors consists of a varying number of objects. We use the probability of survival $p_{s}$ to characterize the uncertainty of target existence. The state of each individual object is described by the vector $x={\left[x,\;\dot{x},\;y,\;\dot{y}\right]}^{T}$, here (x,y) denotes the position of the object and $\left(\dot{x},\;\dot{y}\right)$ denotes the velocity.

The state of each object in the surveillance system evolves according to the Markov transitional density in time, and it is independent of the unknown parameters θ. Here, we use the following motion model:

(44) $$\pi_{k|k-1}\left(x|x_{k-1}\right)=N\left(x;F_{k}x_{k-1},Q_{k}\right)$$

where

(45) $$F_{k}=I_{2}⊗\left[\begin{array}{cc} 1 & T_{k}\\ 0 & 1 \end{array}\right],\;Q_{k}=I_{2}⊗\varpi \left[\begin{array}{cc} \frac{T_{k}^{3}}{3} & \frac{T_{k}^{2}}{2}\\ \frac{T_{k}^{2}}{2} & T_{k} \end{array}\right]$$

and ⊗ is the Kroneker product, $T_{k}=t_{k+1}-t_{k}$ denotes the time interval of the sensors, and ϖ denotes the intensity of process noises.

### 5.2. Experimental Setup

**Figure 1:**
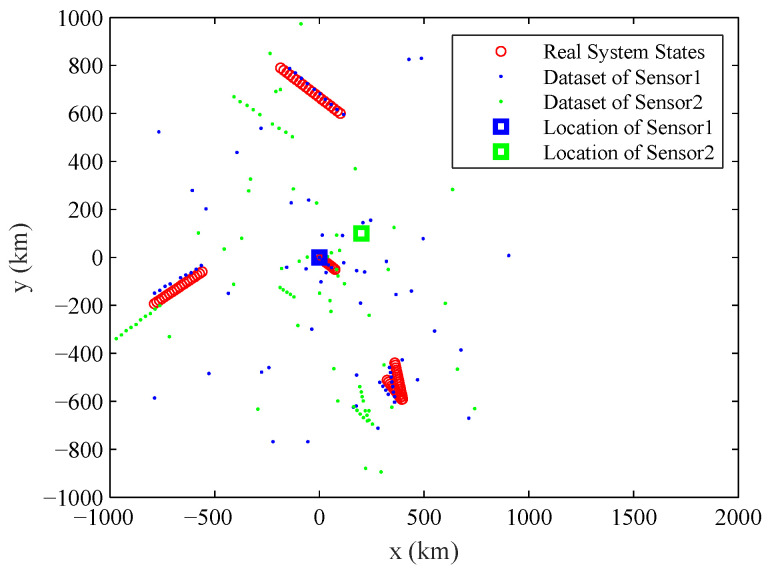
The location of sensors (marked by square), the real states of the studied system which contains multiple objects and the accumulated observation dataset $Z_{1:K}$ for two sensors.

The typical observation dataset sequence $Z_{1:K}$, the real states of the multiple objects and the location of the sensors in the experiment are shown in Figure 1. The number of observation datasets used for estimating parameter is K=20. The number of component objects is varying in the experiment, with $n_{k}=3$ for k=1,2,3,4,5; $n_{k}=5$ for k=6,7,8,9,10; $n_{k}=4$ for k=11,12,13,14,15; and $n_{k}=3$ for k=16,17,18,19,20. The two sensors in the experiment work asynchronously: Sensor 1 provides observation datasets at odd time steps, and Sensor 2 provides observation datasets at even time steps. $T_{k}=1$ s is the time interval between *k* and k−1. The location of two sensors are $\left(x_{1},y_{1}\right)$ = (0 km, 0 km) and $\left(x_{2},y_{2}\right)$ = (200 km, 100 km). The probability of detection is $p_{D}^{\left(1\right)}=p_{D}^{\left(2\right)}=0.98$, the expected numbers of Poisson clutters for two sensors are: $\lambda_{1}=\lambda_{2}=5$, while $c_{1}\left(z|\theta \right)=c_{2}\left(z|\theta \right)$ are uniform distribution over Z=[−π,π]× [0 km, 1000 km]. The standard deviation of observation noises is $\sigma_{\rho }^{\left(1\right)}=\sigma_{\rho }^{\left(2\right)}=$ 1 km and $\sigma_{\eta }^{\left(1\right)}=\sigma_{\eta }^{\left(2\right)}=0.2^{\circ }$. The true systematic errors are $\Delta \eta_{1}=-3^{\circ }$, $\Delta \rho_{1}=$ 10 km, $\Delta \eta_{2}=3^{\circ }$, $\Delta \rho_{2}=-10$ km.

The SMC based implementation of PHD filter uses N=1000 particles per newborn object and persistent object. The probability of object survival is $p_{s}=0.99$. The expected number of object birth is 0.03. The prior distribution $p_{0}\left(\theta \right)$ is a uniform distribution over $\left[-10^{\circ },+10^{\circ }\right]\times \left[-20\;\mathrm{km},+20\;\mathrm{km}\right]\times \left[-10^{\circ },+10^{\circ }\right]\times \left[-20\;\mathrm{km},+20\;\mathrm{km}\right]$. The sample number in the systematic errors space is M=1000. The parameters used in adaptively choosing β are: ϕ=65 and H=0.5M. We set the maximum number of stages by S=10.

### 5.3. Experimental Results

**Figure 2:**
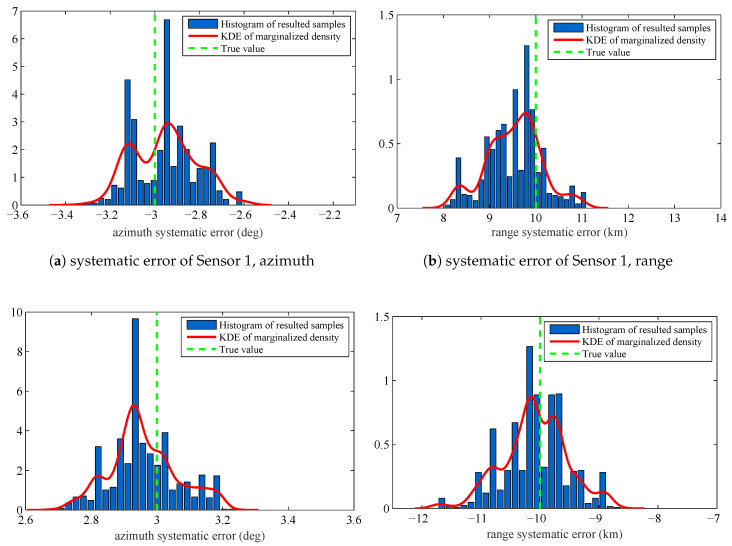
Histograms with distribution fit of the systematic error sample ${\left\{\theta_{j}^{S}\right\}}_{j=1}^{M}$ that are marginalized to $\Delta \eta_{1}$: (**a**) systematic error of Sensor 1, azimuth, $\Delta \rho_{1}$; (**b**) systematic error of Sensor 1, range, $\Delta \eta_{2}$; (**c**) systematic error of Sensor 2, azimuth, $\Delta \rho_{2}$; and (**d**) systematic error of Sensor 2, range.

The estimated systematic errors for the sensors are given in Figure 2. Figure 2 is the histogram with a distribution fit of the resulted sample ${\left\{\theta_{i}^{S}\right\}}_{i=1}^{M}$. Figure 2a–d marginalizes the posterior PDF $p(\theta |Z_{1:K})$ approximated by the resulted samples to one dimension and obtain the estimated azimuth systematic error Δη and range systematic error Δρ for the sensors. The solid lines in each figure are the kernel density estimate (KDE) of the marginal posterior PDF. The vertical dashed lines in each figure are the true values of the sensors’ systematic errors. Since we have obtained the KDE, we use the maximum a posterior (MAP) method to estimate the sensors’ systematic errors. The estimation results are shown in Table 1.

In Figure 2 and Table 1, we can know that the proposed RFS based parameter estimation algorithm can provide the accurate estimated parameters by using the RFS based measurement model and the measurements set that consists of noises, false detections, and missed detections. This is consistent with the first advantage introduced in Section 1.3. Moreover, the proposed algorithm gives the accurate estimated parameters for the stochastic system in which the number of constituent objects (targets) is varying over time. This is consistent with the second advantage introduced in Section 1.3. The obtained experimental results show an outstanding accuracy of the proposed algorithm, although the size of dataset is only 20 scans.

To quantitatively assess the convergence of the algorithm, we adopt the convergence diagnostic named as the estimated potential scale reduction (EPSR). The definition of EPSR is given in [37,38]. The resulted sequence of correction factors β and the EPSR at each stage s=1,…,10 are listed in Table 2. From this table, we can know that the simulated tempering process works effectively. As the stage number increases, the correction factors of each stage increase, and EPSR at each stage decreases, which means that the resulted sample in the simulated tempering process increasingly concentrates in the area of the parameter space characterized by the true likelihood.

**Figure 3:**
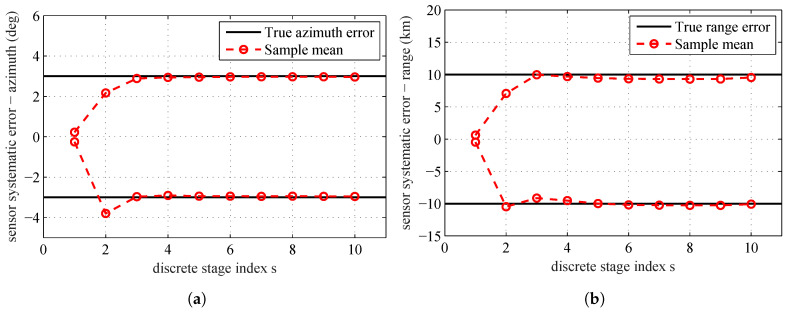
True sensors’ systematic errors and sample mean for all stages: (**a**) sensors’ systematic errors–azimuth; and (**b**) sensors’ systematic errors–range.

The estimates $\theta_{s}$ (the mean of all the particles at each stage) over the simulated tempering stages obtained by running the proposed algorithm are shown in Figure 3. The black solid lines are the true range and azimuth systematic errors of the sensors. The red circles are the sample mean at different tempering stages. From this figure, we can see that, after the fourth stage, the estimation results are very close to the real systematic errors. Since $\Lambda_{s}=\sum_{j=1}^{s}\beta_{j}$ is still less than 1, the estimation process will continue until reaching the maximum stage number S=10.

**Figure 4:**
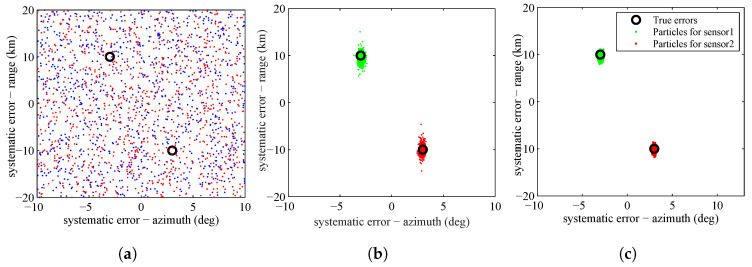
Particle approximation of the posterior PDF of the systematic errors of the sensors at: s=1 (**a**); s=5 (**b**); and s=10 (**c**).

The SMC approximation of the posterior PDF of the sensors’ systematic errors according to Equation (35) at the first stage, fifth stage and tenth stage are shown in Figure 4. The true systematic errors for sensors are represented by black circles. In Figure 4, we can see that, as the simulated tempering process evolves, the particles gradually concentrate on the real systematic errors of the sensors.

### 5.4. System States and Object Number Estimation

**Figure 5:**
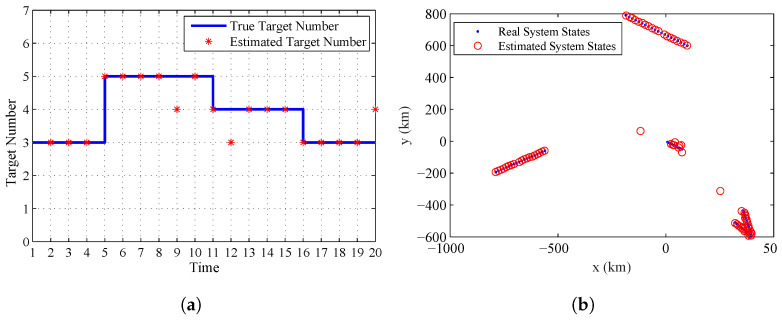
The estimated target number and system states given by the proposed algorithm: (**a**) the real and estimated target number; and (**b**) the real and estimated system states.

As given in Section 1.3, the third advantage of the proposed algorithm is that it can provide the estimate of the system states and the number of targets. Figure 5a gives the real target number contained in the system and the estimated one. Figure 5b gives the real and estimated system states at all the sampling time. From this figure, we can find that the proposed algorithm can accurately estimate the varying number of targets and system states at the final stage by using the estimated systematic errors of sensors.

### 5.5. Comparison with Metropolis Hastings Algorithm

**Figure 6:**
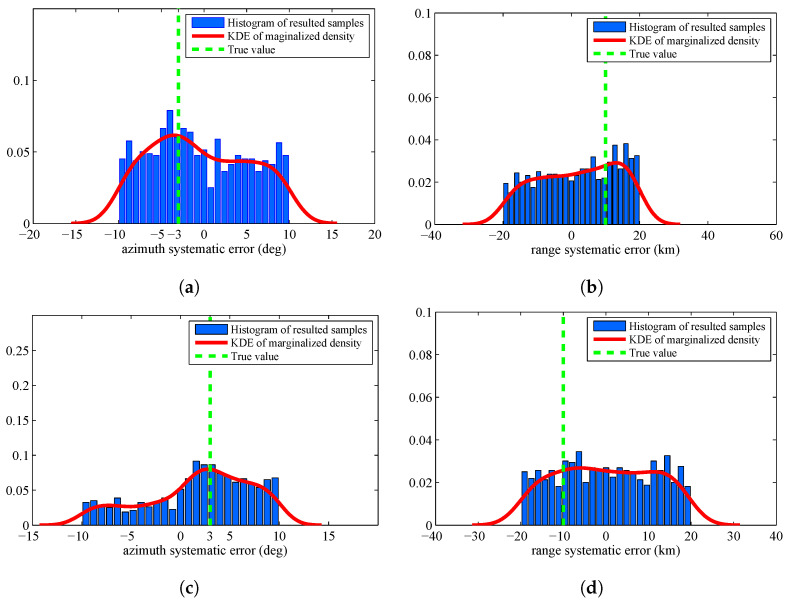
Histograms with distribution fit of the systematic error sample ${\left\{\theta_{j}^{S}\right\}}_{j=1}^{M}$ by using Metropolis Hastings algorithm: (**a**) systematic error of Sensor 1, azimuth; (**b**) systematic error of Sensor 1, range; (**c**) systematic error of Sensor 2, azimuth; and (**d**) systematic error of Sensor 2, range.

There are many probability distributions from which it is impossible to sample directly. The Metropolis Hastings (MH) algorithm is a typical MCMC algorithm that can generate a sequence of random samples from these distributions. Since it is the most widely used method in statistics and in statist physics, we verify the superiority of the proposed algorithm by comparing with MH algorithm. Figure 6 displays the histogram of the resulted samples given by using MH algorithm with the identical experimental setup. From this figure, we can find that the resulted samples given by MH algorithm are more decentralized than the proposed algorithm. The estimated results are shown in Table 3. We can find that the proposed algorithm are more accurate than MH algorithm.

**Figure 7:**
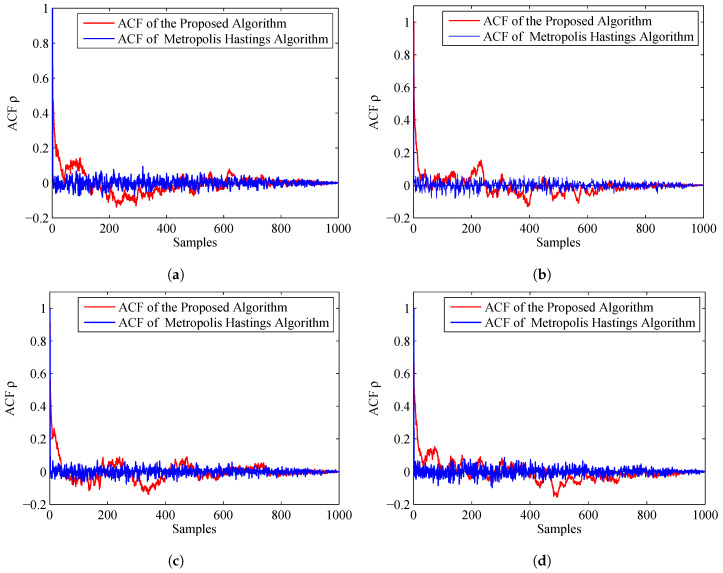
ACF of the proposed algorithm and MH algorithm: (**a**) ACF for Sensor 1, azimuth; (**b**) ACF for Sensor 1, range; (**c**) ACF for Sensor 2, azimuth; and (**d**) ACF for Sensor 2, range.

To compare the accuracy of the importance sampling process, we adapt the evaluation criteria named as autocorrelation function (ACF) [39,40]. According to the fundamentals of MCMC, the samples generated by using MCMC are auto-correlated. Compared with the independent samples, the information content of the samples is relatively reduced. The sample based ACF at lag *k* of a set of samples is defined as follows:

(46) $$\rho_{k}=\frac{\frac{1}{M-k}\sum_{s=1}^{M-k}\left(\theta_{s}-\bar{\theta }\right)\left(\theta_{s+k}-\bar{\theta }\right)}{\frac{1}{M-1}\sum_{s=1}^{M}{\left(\theta_{s}-\bar{\theta }\right)}^{2}}$$

where $\bar{\theta }=\frac{1}{M}\sum_{s=1}^{M}\theta_{s}$ is the Monte Carlo estimate, and $\theta_{s}$ is a sample. From the definition, we can see that, if the ACF dies off to 0 more rapidly, it indicates that the samples are less auto-correlated and more accurate. Figure 7 displays the ACF of the proposed algorithm and MH algorithm. From this figure, we can find that the ACF of the proposed algorithm dies off to 0 more rapidly than the MH algorithm. Thus, the accuracy of the prosed algorithm is much higher.

**Figure 8:**
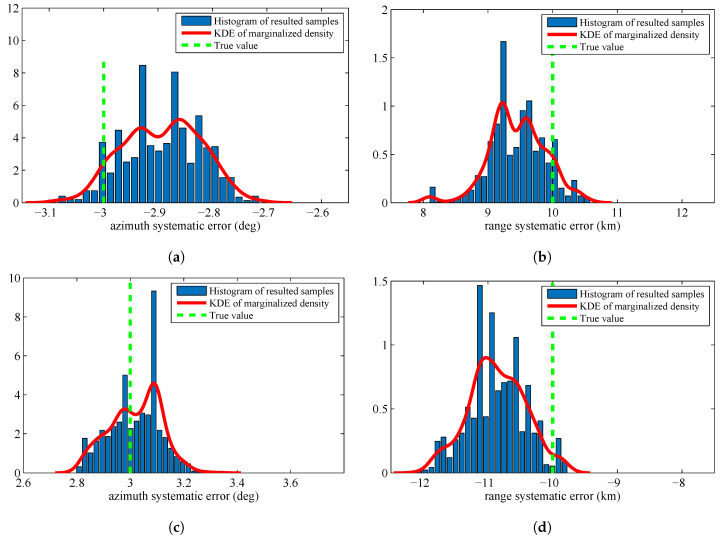
Histograms with distribution fit of the sample ${\left\{\theta_{j}^{S}\right\}}_{j=1}^{M}$ by using the proposed algorithm when the prior is Gaussian distribution: (**a**) systematic error of Sensor 1, azimuth; (**b**) systematic error of Sensor 1, range; (**c**) systematic error of Sensor 2, azimuth; and (**d**) systematic error of Sensor 2, range.

**Figure 9:**
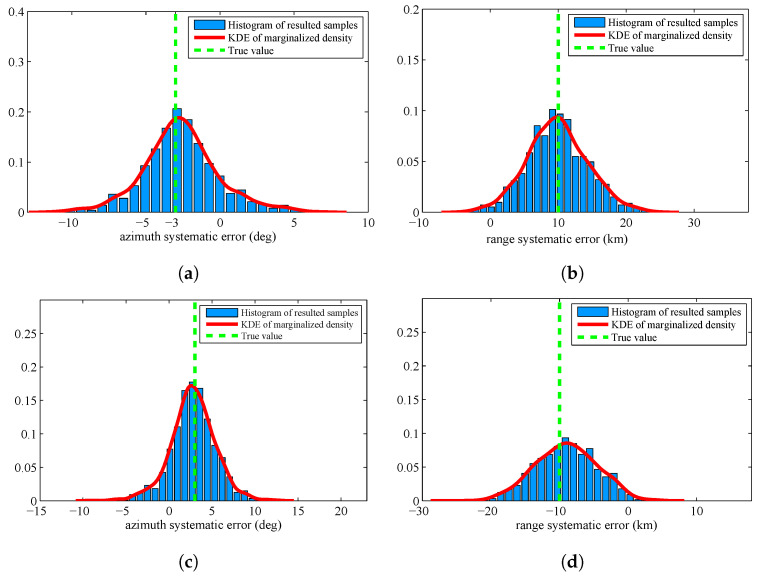
Histograms with distribution fit of the sample ${\left\{\theta_{j}^{S}\right\}}_{j=1}^{M}$ by using MH algorithm when the prior is Gaussian distribution: (**a**) systematic error of Sensor 1, azimuth; (**b**) systematic error of Sensor 1, range; (**c**) systematic error of Sensor 2, azimuth; and (**d**) systematic error of Sensor 2, range.

The former experiments assume that the prior $p_{0}\left(\theta \right)$ is a uniform distribution. To verify the superiority of the proposed algorithm in a more general case, we assume the prior $p_{0}\left(\theta \right)$ is a Gaussian distribution with mean value [−2.5;9.5;2.5;−9.5] and covariance diag [10,20,10,20]. Figure 8 displays the histogram of the resulted samples by using the proposed algorithm. We can find that the resulted KDE densities have deviation by comparing with the former experiment. This is caused by the inaccurate prior distribution. Figure 9 displays the histogram of the resulted samples given by HM algorithm. From this figure, we can find that the resulted samples are still very decentralized and the distribution of samples is heavily depending on the prior distribution.

The estimated results of the two algorithms are given in Table 4. We can find that the accuracy of the proposed algorithm deteriorates slightly, but it is still better than the results of MH algorithm.

**Figure 10:**
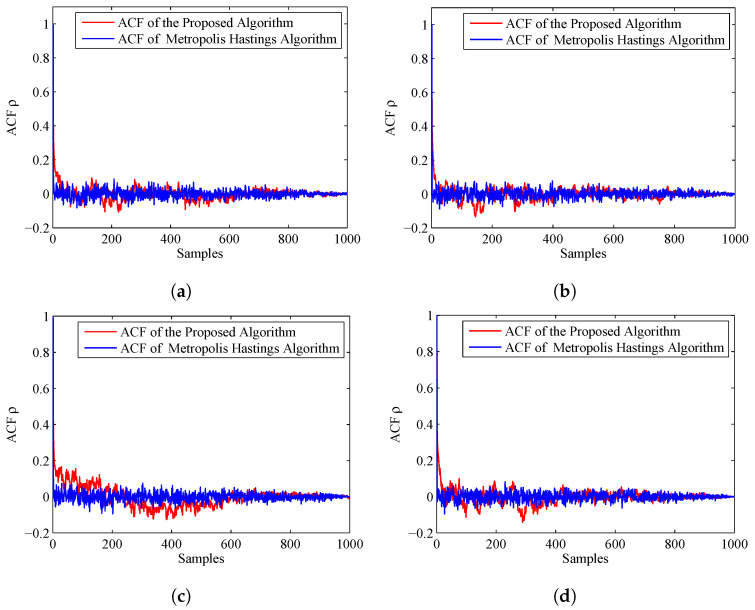
ACF of the proposed algorithm and MH algorithm when the prior is Gaussian distribution: (**a**) ACF for Sensor 1, azimuth; (**b**) ACF for Sensor 1, range; (**c**) ACF for Sensor 2, azimuth; and (**d**) ACF for Sensor 2, range.

The ACF of the two algorithms is given in Figure 10. We can find that the ACF of the proposed algorithm dies off to 0 more slowly than the former experiment, but still more rapidly than the MH algorithm. Thus, the accuracy of the estimated results of the proposed algorithm is a little better than the MH algorithm.

### 5.6. Sensitivity Analysis

In this section, we analyze the sensitivity of the proposed algorithm to a number of important parameters, such as the particle number *N* for PHD filter, the sample number *M* in parameter space as well as the parameter ϕ related to the simulated tempering process.

#### 5.6.1. Particle Number *N*

**Figure 11:**
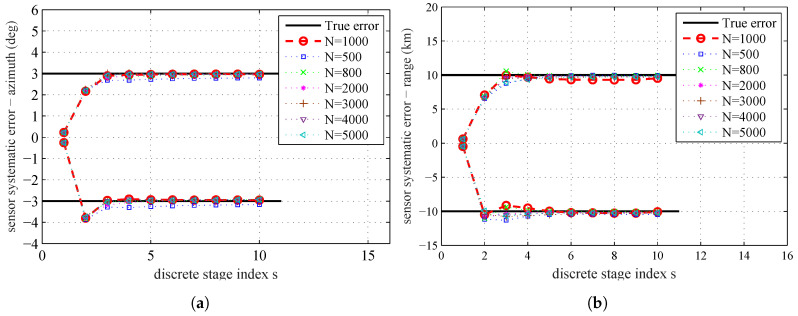
The sample mean for different particle number *N* used by PHD filter: (**a**) sensors’ systematic errors–azimuth; and (**b**) sensors’ systematic errors–range

The PHD filter can provide the estimation of the likelihood $f(Z_{1:K}|\theta )$. Hence, the accuracy of the proposed parameter estimation algorithm may depend on the particle number *N* used by the SMC based PHD filter. The influence of the particle number *N* on the parameter estimation is presented in Figure 11. The estimated systematic errors at the 10th stage for different particle number *N* by using MAP are given in Table 5.

In Figure 11 and Table 5, we can find that the estimation error has a decreasing tendency as *N* increases. With more particles, PHD filter can give more accurate likelihood $f(Z_{1:K}|\theta )$, and thus leads to a better coverage of the parameter space. The plot shows that the decreasing tendency in estimation errors by increasing *N* is not proportional, and when *N* increases to a certain degree, the improvement of estimation accuracy is very small. The result shows that, if *N* increases to a certain degree, it will not significantly affect the parameter estimation results. As the accuracy of the estimation results is restricted by the quality of the model and data used in the parameter estimation process, the estimation results without any errors by continuously increasing *N* is impossible. The computational complexity will also be increased as *N* increases. Therefore, we should achieve a balance between computational complexity and estimation accuracy.

**Figure 12:**
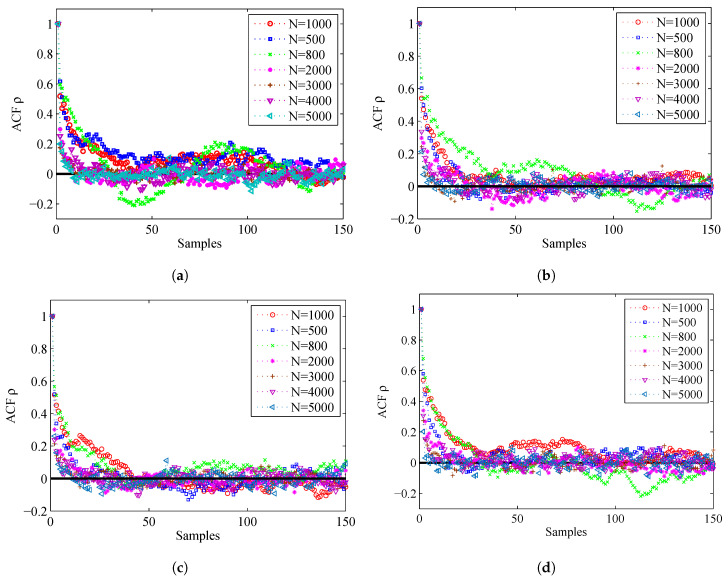
ACF for different particle number *N*: (**a**) ACF for Sensor 1, azimuth; (**b**) ACF for Sensor 1, range; (**c**) ACF for Sensor 2, azimuth; and (**d**) ACF for Sensor 2, range.

Figure 12 shows the ACF by using different values of *N*. For the convenience of analysis, we give the ACF of the first 150 samples. From this figure, we can find that, if *N* is larger, the corresponding ACF will die off to 0 more rapidly, so the accuracy is higher.

Table 6 shows the EPSR at all the stages for different particle number *N* used by the PHD filter. From this table, we can find that, as the number of stage increases, the EPSR of each stage decreases. This means that as the simulated tempering process progresses, the sample increasingly concentrates in the area of the parameter space characterized by the true likelihood. The increase of the particle number *N* seams to have no influence on the convergence of the proposed algorithm.

#### 5.6.2. Sample Number *M*

**Figure 13:**
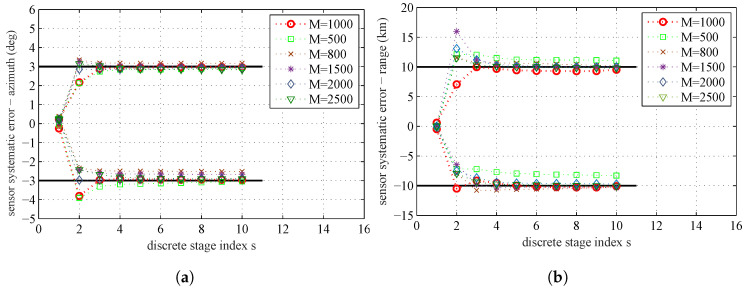
The sample mean for different sample number *M* in the parameter space: (**a**) sensors’ systematic errors–azimuth; and (**b**) sensors’ systematic errors–range.

The sample number *M* in the parameter space may also have influence on the accuracy of parameter estimation. The influence of the sample number *M* on the parameter estimation result is summarized in Figure 13. Table 7 is the MAP estimate at the 10th stage for different *M*. From the plot, we can find that the decreasing tendency in estimation errors by increasing *M* is quite similar to the result caused by increasing *N*. If *M* increases to a certain degree, the improvement of estimation accuracy is very small. There also needs a balance between computational complexity and estimation accuracy.

The EPSR of different sample size *M* is given in Table 8. From this table, we can find that the increase of *M* will result in the increase of EPSR, which means that the convergence of the proposed algorithm will be increased.

#### 5.6.3. Simulated Tempering Parameter

**Figure 14:**
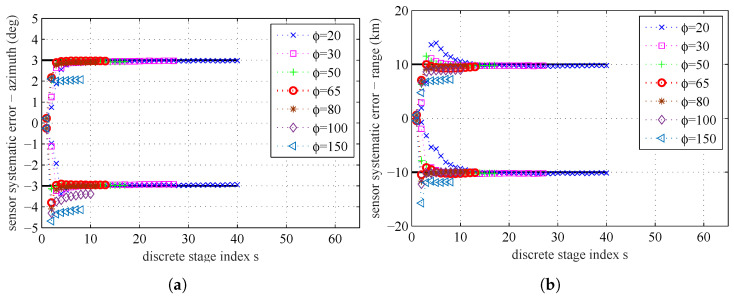
The sample mean for different ϕ: (**a**) sensors’ systematic errors, azimuth; and (**b**) sensors’ systematic errors, range.

**Figure 15:**
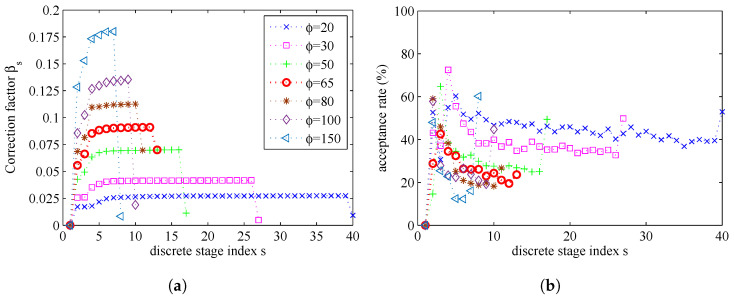
The relationship between correction factors, acceptance rates and ϕ: (**a**) correction factors; and (**b**) acceptance rates.

By setting the maximum stage number to be S=∞, the influence of the parameter ϕ on the parameter estimation results is presented in Figure 14 and Figure 15. In Figure 14, we can find that a smaller ϕ will need more stages and get more accurate estimation result. If ϕ is bigger than a certain degree, the number of stages will be very small, and the estimation errors will be bigger. In Figure 15b, we can find that, if a lower value of parameter ϕ is used, the MCMC acceptance rate will be increased. In Figure 15a, we can find that, if a lower value of parameter ϕ is used, the number of stages *S* will be increased, because the increments of correction factors $\beta_{s}$ become smaller.

The computational complexity of simulated tempering based importance sampling is determined by the number of stages *S* and the correction factors $\beta_{1},\cdots ,\beta_{S}$. To reduce the computational complexity, we tend to use a small value of *S*. However, at the same time, if we choose a small *S*, the sequential intermediate distribution $\vartheta_{s}$ will vary rapidly. There is a balance between the number of stages (related to the computational complexity) and the estimation accuracy.

We give the estimated systematic errors at the final stage for different ϕ in Table 9. It seems that, if ϕ decreases, the stage number will increase and the estimated results will be more accurate.

## 6. Conclusions

We have presented the proposed RFS based parameter estimation algorithm to overcome some disadvantages of the vector based Bayesian algorithms. We also detail how to implement this algorithm by using the SMC based PHD filter and simulated tempering based importance sampling. By applying the SMC based PHD filter, the proposed algorithm successfully extends the application of parameter estimation from the single object system to the stochastic system which consists of a varying number of constituent objects. Without any assumptions under ideal conditions, it can fit the dynamic nature of the studied system and the detection uncertainty of the measurement system very well. It can be successfully used for identification of the stochastic systems which consist of a varying number of constituent objects. It can also be used for parameter estimation problem where the observation dataset is affected by false detections, missed detections and noises.

## Figures and Tables

**Table 1:** True and estimated sensors’ systematic errors.

	$\Delta \eta_{1}$ (deg)	$\Delta \rho_{1}$ (km)	$\Delta \eta_{2}$ (deg)	$\Delta \rho_{2}$ (km)
True values	−3	10	3	−10
Estimated values	−2.9399	9.9790	2.9418	−10.0558

**Table 2:** Correction factors and EPSR during simulated tempering.

s	1	2	3	4	5	6	7	8	9	10
$\beta_{s}$	0.0557	0.0663	0.0854	0.0883	0.0896	0.0903	0.0906	0.0908	0.0910	0.0700
EPSR	1.0043	0.9492	0.8862	0.8731	0.8680	0.8673	0.8671	0.8670	0.8669	0.8667

**Table 3:** True and estimated sensors’ systematic errors by using two algorithms.

	$\Delta \eta_{1}$ (deg)	$\Delta \rho_{1}$ (km)	$\Delta \eta_{2}$ (deg)	$\Delta \rho_{2}$ (km)
True values	−3	10	3	−10
Estimated values by proposed algorithm	−2.9399	9.9790	2.9418	−10.0558
Estimated values by MH algorithm	−3.6075	13.1868	2.7403	−5.9794

**Table 4:** True and estimated sensors’ systematic errors for Gaussian distribution.

	$\Delta \eta_{1}$ (deg)	$\Delta \rho_{1}$ (km)	$\Delta \eta_{2}$ (deg)	$\Delta \rho_{2}$ (km)
True values	−3	10	3	−10
Estimated values by proposed algorithm	−2.8619	9.2276	3.0909	−11.0458
Estimated values by MH algorithm	−2.7219	9.7624	2.4841	−8.8048

**Table 5:** Estimated systematic errors at stage *s* = 10 for different particle number *N*.

*N*	500	800	1000	2000	3000	4000	5000
$\Delta \eta_{1}$ (deg)	3.1676	−2.9190	−2.9399	−2.9443	−2.9465	−2.9415	−2.9418
$\Delta \rho_{1}$ (km)	9.9420	9.9475	9.9790	9.9776	9.9874	9.9828	9.9813
$\Delta \eta_{2}$ (deg)	2.7879	2.9364	2.9418	2.9739	3.0019	2.9791	2.9827
$\Delta \rho_{2}$ (km)	−10.4852	−10.0786	−10.0558	−10.0259	−10.0569	−10.0507	−10.0481

**Table 6:** EPSR for different particle number *N*.

*N*	500	800	1000	2000	3000	4000	5000
*s* = 1	1.0043	1.0043	1.0043	1.0043	1.0043	1.0043	1.0043
*s* = 2	0.9482	0.9447	0.9492	0.9426	0.9439	0.9344	0.9440
*s* = 3	0.8778	0.8765	0.8862	0.8756	0.8726	0.8780	0.8744
*s* = 4	0.8694	0.8692	0.8731	0.8684	0.8679	0.8691	0.8687
*s* = 5	0.8667	0.8674	0.8680	0.8674	0.8673	0.8677	0.8674
*s* = 6	0.8663	0.8670	0.8673	0.8672	0.8671	0.8672	0.8671
*s* = 7	0.8662	0.8670	0.8671	0.8670	0.8671	0.8671	0.8670
*s* = 8	0.8662	0.8670	0.8670	0.8668	0.8670	0.8671	0.8670
*s* = 9	0.8661	0.8669	0.8669	0.8668	0.8669	0.8670	0.8668
*s* = 10	0.8661	0.8668	0.8667	0.8668	0.8667	0.8669	0.8668

**Table 7:** Estimated systematic errors at stage s=10 for different sample numbers *M*.

M	500	800	1000	1500	2000	2500
$\Delta \eta_{1}$ (deg)	−3.0428	−2.5120	−2.9399	−2.9704	−2.9844	−2.9816
$\Delta \rho_{1}$ (km)	11.4438	9.7102	9.9790	9.9889	10.0056	10.0567
$\Delta \eta_{2}$ (deg)	3.2298	2.8761	2.9418	3.0196	2.9482	2.9519
$\Delta \rho_{2}$ (km)	−8.2334	−10.3842	−10.0558	−10.2542	−9.8020	−10.0881

**Table 8:** EPSR of different sample size *M*.

*M*	500	800	1000	1500	2000	2500
*s* = 1	1.0039	0.9905	1.0043	1.0030	1.0661	1.0560
*s* = 2	0.9348	0.9111	0.9492	0.9621	0.9852	0.9933
*s* = 3	0.8784	0.8681	0.8862	0.9028	0.8929	0.9016
*s* = 4	0.8700	0.8671	0.8731	0.8774	0.8780	0.8791
*s* = 5	0.8680	0.8668	0.8680	0.8772	0.8773	0.8779
*s* = 6	0.8674	0.8667	0.8673	0.8671	0.8671	0.8673
*s* = 7	0.8671	0.8666	0.8671	0.8668	0.8669	0.8671
*s* = 8	0.8666	0.8666	0.8670	0.8667	0.8668	0.8670
*s* = 9	0.8665	0.8665	0.8669	0.8666	0.8667	0.8668
*s* = 10	0.8665	0.8665	0.8667	0.8666	0.8666	0.8667

**Table 9:** Estimated systematic errors at the final stage for different ϕ.

ϕ	20	30	50	65	80	100	150
Maximum stage number	40	27	17	13	11	10	8
$\Delta \eta_{1}$ (deg)	−2.9850	−2.9832	−2.9717	−2.9399	−3.0010	−3.3901	−4.1380
$\Delta \rho_{1}$ (km)	9.9788	9.9784	9.9484	9.9790	9.7276	8.9416	7.2124
$\Delta \eta_{2}$ (deg)	2.9770	2.9701	2.9295	2.9418	2.8938	2.6447	2.0738
$\Delta \rho_{2}$ (km)	−9.9075	−10.0889	−10.1899	−10.0558	−9.9267	−10.4167	−11.8311

## Supplementary Materials

The supplementary files are the source codes for the experiments of this paper. The related experimental results are also given. The files are available online at http://www.mdpi.com/1099-4300/20/8/569/s1.

## Abbreviations

FISST	Finite Set Statistics
KDE	Kernel Density Estimate
MCMC	Markov Chain Monte Carlo
ML	Maximum Likelihood
PDF	Probability Density Function
PF	Particle Filter
PHD	Probability Hypothesis Density
RFS	Random Finite Set
SMC	Sequential Monte Carlo
